# Systematic-Narrative Hybrid Literature Review: Crosstalk between Gastrointestinal Renin–Angiotensin and Dopaminergic Systems in the Regulation of Intestinal Permeability by Tight Junctions

**DOI:** 10.3390/ijms25105566

**Published:** 2024-05-20

**Authors:** Nadia Khan, Magdalena Kurnik-Łucka, Gniewomir Latacz, Krzysztof Gil

**Affiliations:** 1 Faculty of Medicine, Department of Pathophysiology, Jagiellonian University Medical College, Czysta 18, 31-121 Krakow, Poland; 2 Faculty of Pharmacy, Department of Technology and Biotechnology of Drugs, Jagiellonian University Medical College, Medyczna 9, 31-008 Krakow, Poland

**Keywords:** ACE2, dopamine, DDC, DOPA decarboxylase, angiotensin (1-7), Na^+^/K^+^-ATPase, B^0^AT1, tight junctions, gastrointestinal, leaky gut

## Abstract

In the first part of this article, the role of intestinal epithelial tight junctions (TJs), together with gastrointestinal dopaminergic and renin–angiotensin systems, are narratively reviewed to provide sufficient background. In the second part, the current experimental data on the interplay between gastrointestinal (GI) dopaminergic and renin–angiotensin systems in the regulation of intestinal epithelial permeability are reviewed in a systematic manner using the PRISMA methodology. Experimental data confirmed the copresence of DOPA decarboxylase (DDC) and angiotensin converting enzyme 2 (ACE2) in human and rodent enterocytes. The intestinal barrier structure and integrity can be altered by angiotensin (1-7) and dopamine (DA). Both renin–angiotensin and dopaminergic systems influence intestinal Na^+^/K^+^-ATPase activity, thus maintaining electrolyte and nutritional homeostasis. The colocalization of B^0^AT1 and ACE2 indicates the direct role of the renin–angiotensin system in amino acid absorption. Yet, more studies are needed to thoroughly define the structural and functional interaction between TJ-associated proteins and GI renin–angiotensin and dopaminergic systems.

## 1. Introduction

**Figure 1:**
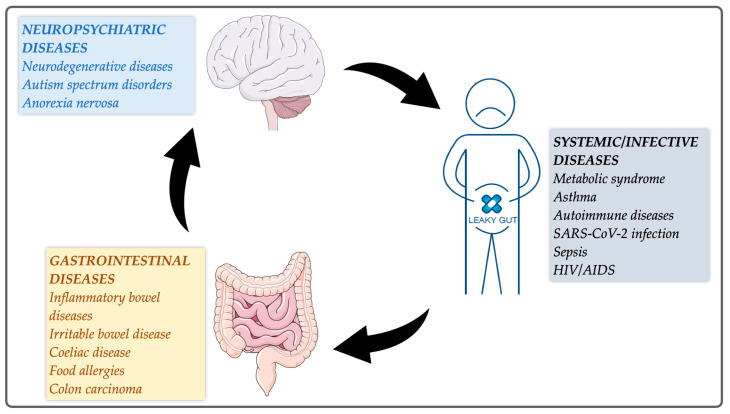
Examples of gastrointestinal and extraintestinal diseases associated with intestinal barrier defects known as “leaky gut” [2,3,4,5,6,7]. The figure was created using Servier Medical Art licensed under CC BY 4.0.

The intestinal mucosal barrier separates the intermilieu of the body from the external environment by means of physical, biochemical, and immune elements. The semipermeable intestinal epithelium is an essential component of the mucosal barrier and maintains local homeostasis by acting as a protective barrier and selective filter, as well as serves as an essential modulator of the immune system [1]. The intestinal epithelium mediates permeability via two major routes: transepithelial/transcellular and paracellular. Transcellular permeability is a transport through the epithelial cells that is mainly regulated by endocytosis, passive diffusion, or selective transporters for amino acids, electrolytes, short-chain fatty acids, sugars, and any large antigenic molecules. Paracellular permeability, preferred by small hydrophilic molecules (<600 Da), is a transport in-between for epithelial cells regulated by transcellular complexes localized at the apical–basolateral membrane junctions with the pore and the leak pathways [2,3]. Disruption of the intestinal mucosal barrier has been hypothesized to be a primary trigger for the development of gastrointestinal (GI) [4,5], systemic inflammatory, and neurological diseases [2,3,6,7]. Examples of such disorders are given in Figure 1. Recently, for example, it was reported that COVID-19 patients were characterized by significantly elevated levels of circulating zonulin family peptides that regulate intestinal permeability in serum [8]. Still, it remains unclear whether intestinal barrier loss can be a consequence of dysfunctional intercellular junctions or a direct epithelial cell damage or both [2].

So far, studies have shown important interactions between the local renin–angiotensin system (RAS) and dopaminergic (DAergic) system in cardiorenal syndrome [9,10,11], neurodegeneration [12,13], and inflammation [14]. The functional components of both the RAS and DAergic system are expressed in the GI mucosa. It was long ago that Stevens et al. (1988) suggested that high doses of orally administered ACE inhibitors may affect intestinal epithelial function [15]. Yet, the understanding of the GI mucosal DAergic system is less apparent.

Thus, the aim of this systematic narrative hybrid literature review is twofold. In the first part, the role of tight junctions in the regulation of intestinal epithelial permeability is briefly and narratively reviewed to provide sufficient background. Despite the complexity of tight junctions, their understanding has vastly expanded; thus, complete functional description of all tight junction-associated proteins is inarguably beyond the scope of this article. Furthermore, the GI DAergic system and RAS are described in the context of their localization. In the second part, the current experimental data, including morphological and functional correlates on the interplay between the GI DAergic and renin–angiotensin systems in the regulation of intestinal epithelial permeability is reviewed in a systematic manner using the PRISMA methodology [16].

## 2. Materials and Methods

**Figure 2:**
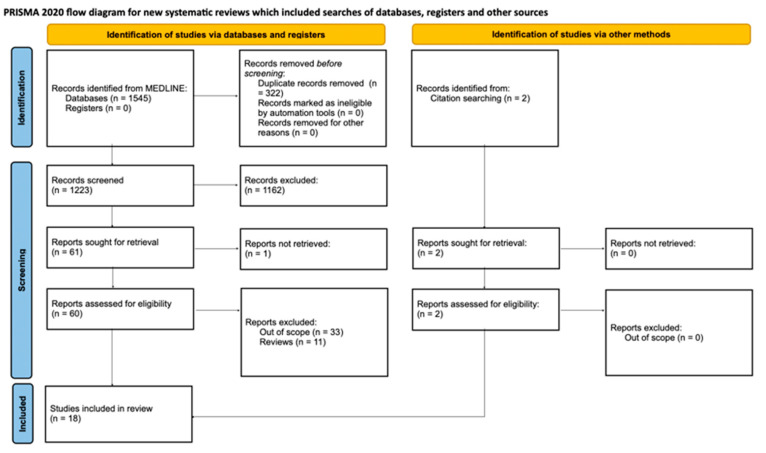
The flow diagram prepared according to PRISMA 2020 guidelines [16].

A systematic search was conducted according to the Preferred Reporting Items for Systematic reviews and Meta-Analyses (PRISMA) methodology [16]. The MEDLINE database was searched from its earliest records, and a reference tool called *My bibliography* (National Library of Medicine) was used to manage citations. The search was performed between 16 January and 3 February 2024. The following search terms were applied for the study: ACE, ACE2, angiotensin, AT1, AT2, B(0)AT1/2, DDC, D1/2/3/4/5 receptor, dipeptidyl carboxypeptidase, dopamin*, epithelial permeability, intestin*, l-dopa, levodopa, MasR, Mas receptor, Na-K-ATPase, neprylysin, occludin, renin, tight junction, tyrosine hydroxylase, and zonulin. All duplicates were removed, and all articles were independently screened. The criteria for eligibility consisted of the following: English language, original article type (including case reports), full text availability, and adherence to the research topic (only mammalian models, human studies, and ex vivo models were included, while cell line-based studies were excluded). Additionally, a reference search was performed, and a functionality of PubMed—“*similar articles*”—was used. Articles were evaluated via paper title, abstract, and content in accordance with the agreement of the works with our inclusion criteria. The final list of full texts was double-checked, and the data were extracted independently by all investigators blinded to each other. The PRISMA flow diagram is illustrated in Figure 2.

## 3. Results

### 3.1. Part 1—A Narrative Review

#### 3.1.1. What Are the Morphological and Functional Characteristics of Intestinal Epithelial Cells in the Context of Intestinal Permeability?

Intestinal epithelial cells (IECs) line the luminal surface of the intestinal epithelium and are arranged throughout the small and large bowel in a continuous manner. IECs are capable of recognizing bacterial-derived molecules, ingesting bacteria, and neutralizing bacterial toxins [7]. The aged IECs undergo apoptosis, are removed from the villous tip into the intestinal lumen, and are continuously replaced every 4–5 days by new IECs produced by stem cells located in the middle of the crypts [17]. The stem cells have a high proliferative rate with embryonic cell-like features. A large nucleus compartment with diffuse chromatin and scant cytoplasm with few small organelles are characteristic morphological features of those stem cells [18,19]. Small intestine enterocytes are simple columnar epithelial cells with apical microvilli that immensely increase their absorptive surface and represent the most prominent cell type among IECs (around 80%). The principal function of the adult colonocytes (also simple columnar epithelial cells) is to absorb water and salt, and villi are absent [17]. Apart from enterocytes and colonocytes, various differentiated and specialized IECs are found within the intestinal epithelium: secretory cells such as goblet cells, enteroendocrine cells, Paneth cells, chemosensory tuft cells, and M (microfold or membranous) cells. It is important to note that Paneth cells, which intercalate with stem cells at the bottom of crypts and M cells overlaying Peyer’s patches, are unique to the small intestine. All of these above-mentioned cells can develop specialized functions and thus can be further classified into subsets [19].

It was Farquhar and Palade (1963) who demonstrated that there are small areas of fusion between adjacent epithelial cells. These areas were described as a tripartite junctional complex structure in the epithelia of various glands and cavity organs and were predominantly visualized in the mucosal cells of intestines and glandular epithelia of stomach [20]. These tiny intercellular spaces between adjacent cells were initially described as ‘intercellular spaces’, ‘intercellular connections’, and ‘intercellular junctions’. These junctions are highly specialized multilayered membranes and are classified into 3 different types: tight junctions (TJs or zonula occludens), desmosomes (DMs or macula adherens), and adherent junctions (AJs or zonula adherens) [20,21]. TJs, which are the most apical junctions, create a watertight sealing between the intercellular epithelial spaces and form a barrier to limit the paracellular pathway of solutes [22,23,24]. The TJs also develop polarization by forming a boundary between apical and basolateral membrane [24]. The DMs and AJs are intercellular basolateral contacts that connect neighboring epithelial cells together via dynamic actin cytoskeleton and intermediate filaments in the cytoplasm, respectively. Although both junctional complexes are essential for establishing mechanical linkages between cells, they also have indispensable roles in cell–cell contact formation, stabilization, and maturation [25]. Studies have reported that both TJ and AJ complexes form an intramembrane diffusion fence that regulates polarization, cell proliferation, and differentiation [22,26,27].

The IECs contribute to the formation of TJs between neighboring cells, where they regulate barrier function to maintain gut homeostasis. TJs and their associated proteins play a critical role in the intestinal barrier function maintenance [28]. The proteins of TJs are not static intercellular barriers but highly dynamic proteins that are constantly remodeled due to connections with various intrinsic and extrinsic stimuli, such as, for example, flow of water molecules, ions, food residues, and microorganisms or injury [7,29]. TJs consist of two classes of proteins: peripheral (or plaque) membrane and integral transmembrane proteins. Plaque proteins are required for the coordination of signals coming from the plasma (cell) membrane, and transmembrane proteins directly interact with the actin in cytoskeleton and with other submembrane proteins. Zonula occludens proteins are the most recognized and well studied peripheral membrane proteins. The integral transmembrane proteins are classified into at least the following: occludin, claudins, junctional adhesion molecules, and tricellulin. The major proteins associated with TJs expressed in the intestinal epithelium are listed in Table 1. The integrity and barrier function of TJs are dynamically regulated by the interaction between integral transmembrane and peripheral proteins [28]. The synthesis, distribution, and phosphorylation of these TJ proteins define their barrier function. Several signaling pathways, including protein kinase C, A, and G; phosphatase; Rho; myosin light-chain kinase (MYLK or MLCK); mitogen activated-kinase (MAPK); AMP-activated protein kinase (AMPK); and PI3K/Akt, are involved in the continuous regulation of TJ proteins [23,28,30].

#### 3.1.2. How TJs Impact Intestinal Permeability?

Zonula occludens-1 (zonulin-1, ZO-1) was discovered as the first intracellular component of TJs in 1986 [39]. Subsequently, occludin was identified as the first transmembrane protein of TJs in 1993 [40]. Phosphorylated occludin controls TJ stability and permeability [41], and the interaction between occludin and ZO-1 is critical for TJ integrity [40]. Studies showed that the phosphorylation of occludens due to oxidative stress caused disruption in barrier function [41,42,43]. In vitro, the ubiquitination and phosphorylation of occludin were required for disruption of the ZO-1 protein, the increase in permeability of epithelial cells, and the destabilization of bonds between TJs and enterocytes [42]. It was also reported that a single deletion of occludin and tricellulin proteins should only cause minimal changes in the structure, integrity, and permeability function of the TJ, whereas double deletion of these two proteins should alter their morphology by reducing the crosslinks between the junction proteins and attenuate barrier function by enhancing the permeability of ions, fluid, and small molecules, thus suggesting the significance of occludin and tricellulin in the intestinal TJ’s permeability and homeostasis [44,45].

Occludin, tricellulin, ZO-1, and perijunctional actomyosin regulate the so-called leak pathway, thus allowing the flux of molecules with diameters of up to ~12.5 nm regardless of their charge [2]. Claudin transmembrane proteins have dual functions, thus acting as a “tight or sealing” or as “leaky or pore-forming” claudins. They have different charge selectivity, and therefore, their paracellular properties are based on the composition of amino acids in their domains [46]. The TJ’s pore- or channel-forming claudins, also referred to as paracellular tight junction channels, exhibit biophysical properties similar to traditional ion channels; for instance, claudin-2 [47], -15, [48], and -16 (also known as paracellin) [49] facilitate paracellular cation permeability. The substitution of a single amino acid in claudins can alter selectivity and reduce the permeability for cations [33]. Claudin-2 and claudin-12 were reported to play a role in the absorption of calcium through independent pores via a paracellular transport mechanism in the colonic epithelium. The double knockout of claudin-2/-12 greatly decreased permeability and calcium homeostasis in mice intestine when compared with the single deletion of a protein, thus indicating that claudin-2 and -12 are important constituents of paracellular calcium transport in intestines [50]. In the intestinal mucosa of young and adult mice, claudin-2 and -15 were found to regulate monovalent ion permeability, particularly for sodium ions. In the absence of paracellular sodium transport, transcellular transport rapidly depletes luminal sodium ion concentration and stops nutrient cotransport across the apical brush border. Thus, the flux across claudin-2 and claudin-15 channels allows sodium ions efflux from the lamina propria to the lumen [2]. The double knockout of these two proteins reduced the uptake of nutrients, caused malabsorption, and induced death in mice [51].

Junctional adhesion molecules (JAMs) can exhibit two different types of cell-to-cell interactions: homophilic and heterophilic. JAMs in homophilic interaction actively express adhesive contacts, i.e., the adhesion molecule on one cell interacts with identical molecules on the other cell. However, in the heterophilic interaction, the adhesion molecule from one cell functions as a receptor for the other molecule (which acts as a ligand) on the other cell [52,53,54]. Intestinal permeability and leucocyte migration depends on JAM immunoglobulin superfamily proteins. The complete knockout of the JAM-A protein in mice showed enhanced leucocyte infiltration, increased permeability to dextran, decreased transepithelial electrical resistance (TEER), lymphoid accumulation, higher myeloperoxidase activity in the colon, and the downregulation of barrier function in intestinal epithelial cells, thus indicating the importance of JAM-A in the assembly of TJs and permeability. In addition, the deletion of the JAM-A protein in knockout mice also caused severe colonic injury and inflammation when compared to wild type control mice [55]. Furthermore, JAM-A-deficient mice also showed increased epithelial cell proliferation in colitis by interacting with tumor suppressor NF2, thus indicating the crucial role of JAM-A in intestinal homeostasis by attenuating permeability function and cell–cell contact in the intestine [56].

#### 3.1.3. Are Intestinal Epithelial Na^+^/K^+^ATPase and Tight Junctions Structurally and Functionally Interconnected?

The Na^+^/K^+^ ATPase of mammalian epithelial cells, also known as the sodium–potassium pump, is the key transporter responsible for the maintenance of intestinal osmotic homeostasis. The pump also provides an essential gradient to drive secondary transport and is involved in various signaling pathways related to cell adhesion, cell proliferation, cell cycle, and fibrosis [57,58,59]. Under physiological conditions, the Na^+^/K^+^ ATPase is localized in the basolateral membrane domain of intestinal epithelial cells and maintains an electrochemical gradient by transferring three sodium ions out and two potassium ions into the cell, and at the same time, it uses one molecule of ATP per cycle [60]. The enzyme maintains a favorable transcellular sodium gradient, which is essential for the proper activity of brush border membrane Na^+^-coupled co-/ countertransporters and ion channels and thus enables the absorption and transport of various nutrient macromolecules, including glucose, amino acids, vitamins, adenosine nucleic acids, and other ions across the epithelium [61,62,63]. The Na^+^/K^+^ ATPase has three subunits, namely alpha (α) and beta (β) subunits present in equimolar amounts and a gamma (γ, ~7 kDa) regulatory subunit [64,65]. The *α* subunit (~110 kDa) is considered to be the catalytic subunit of the enzyme and holds ATP, Na^+^, K^+^, Mg^2+^, and ouabain binding sites [66,67]. The highly glycosylated *β* subunit (~40–60 kDa) is critical for the structural maturation of the holoenzyme and its function [58,62,68]. The enzyme is regulated by intrinsic (kinases and phosphatases) and extrinsic (inflammatory mediators or endocrine signals) factors under physiological and/or pathophysiological conditions [69,70].

The reduced synthesis of Na^+^/K^+^ ATPase was observed, for instance, in animal models of ileal inflammation [71,72,73]. According to in vitro studies on polarized epithelial Madin–Darby canine kidney (MDCK) cell line, an inhibition of the enzymatic activity of the Na^+^/K^+^ ATPase membrane by potassium depletion or ouabain treatment inhibited the formation of TJs and desmosomes, and the cells remained nonpolarized [74]. In the Caco-2 cells monolayer, protein phosphatases 2A and 1, which can form a direct complex with the Na^+^/K^+^ATPase [75], interacted with occludin and negatively regulated the assembly of TJs by adjusting the phosphorylation status of occludin [76]. What is more, the enhanced activity of serine/threonine protein phosphatase-2A (PP2A) was associated with the dephosphorylation of occludin, claudin-1, ZO-1, and enhanced paracellular permeability in MDCK cells [77]. Furthermore, an inhibition of the Na^+^/K^+^ ATPase transportive function inhibited PPA2 activity, which induced the hyperphosphorylation of occludin and prevented the complete formation of functional TJ strands in the human pancreatic epithelial cell line HPAF-II. This noncoordinated assembly of TJ strands altered the polarization of these epithelial cells, thus leading to an increase in the permeability of ionic and nonionic solutes [78]. Overall, the intestinal epithelial Na^+^/K^+^ ATPase and tight junctions should indeed be structurally and functionally interconnected, yet direct data from intestinal cell lines, primary cultures or animal models, are limited and immensely incomplete.

#### 3.1.4. What Are the Key Components of the Renin–Angiotensin System in the Gastrointestinal Tract?

The history of the renin–angiotensin system (RAS) originated with the studies by Tigerstedt and Bergman in 1898 [79]. The RAS is a coordinated hormonal cascade predominantly recognized for its pronounced endocrine role in the control of peripheral vascular resistance and fluid homeostasis, and thus especially in the context of cardiovascular and renal physiology and pathophysiology [80,81]. Yet, due to the accumulating evidence, the concept of “local” or “tissue” RAS has already been introduced [81]. The first step of the RAS involves the cleavage of angiotensinogen into angiotensin (Ang) I (1-10) by renin, which is an aspartyl protease (EC 3.4.23.15) that belongs to the protein family peptidase A1. So far, an active renin is known to originate in the juxtaglomerular cells of the kidneys, although extrarenal sources of (pro)renin are known [82,83]. Further on, a decapeptide Ang I is converted into either an octapeptide Ang II (1-8) by the angiotensin-converting enzyme (ACE; EC 3.4.17.23), a nanopeptide Ang (1-9) by the angiotensin-converting enzyme 2 (ACE2; EC 3.4.17.23), or a heptapeptide Ang (1-7) by neprylysin (a neural endopeptidase; EC 3.4.24.11). Ang (1-9) can be also converted into Ang (1-7) by both ACE2 and neprylysin. Ang II is a pivotal product of the so-called classical RAS and is rapidly degraded by different aminopeptidases (AP) into a heptapeptide Ang III (2-8) and a hexapeptide Ang IV (3-8). Interestingly, the Ang II (1-8) of the classical RAS also enters the nonclassical RAS system, where it can be cleaved by ACE2 to Ang (1-7) [81]. ACE activates phospholipase C by raising the intracellular calcium level and activating protein kinase C, and it inhibits adenylate cyclase and activates tyrosine kinase [84]. ACE2 has a 400-fold stronger affinity for Ang II than Ang I, and thus, the primary function of ACE2 is the formation of Ang (1-7) [85]. And ACE2 also acts as a downregulator of the RAS [84,85]. It is noteworthy that the highest tissue concentrations of ACE and ACE2 mRNA in the human body have been found in the duodenum, terminal ileum, and colon [86,87]. Ang II action is mediated via G protein-coupled receptors having seven membrane-spanning domains of two subtypes: angiotensin type 1 (AT1R) and type 2 (AT2R) receptors, which can also directly interact between themselves [81]. In general, AT2R counterregulates the action of AT1R [88]. The existence of an additional receptor AT4R has also been reported [81]. Furthermore, Ang (1-7) action is mediated via a G protein-coupled Mas receptor (MasR) [89].

The first reports in relation to the RAS system and the GI tract date back to the 1960s of the past century [90,91]. Now, the GI RAS is recognized to regulate/coordinate such functions as intestinal mucosal protection, motility, absorption and digestion, water–electrolyte balance, glucose transport, inflammatory response, fibrosis and tissue remodeling, aging, and carcinogenesis (these functions are comprehensively reviewed in [92,93,94,95]). Table 2 lists the key RAS components expressed in the healthy human and rodent GI tract.

#### 3.1.5. What Are the Key Components of the Dopaminergic System in the Gastrointestinal Tract?

The dopamine (DA) molecule consists of a catechol structure (a benzene ring with two hydroxyl side groups) with one amine group attached via an ethyl chain; it is a monoamine actively present in the body and involved in the regulation of many physiological functions centrally and peripherally [122]. In the periphery, the dopaminergic (DAergic) system is mainly found in mesenteric organs [123]. A DA molecule can be synthesized from the nonessential amino acid L-tyrosine (4-hydroxyphenylalanine, TYR) or from the essential amino acid L-phenylalanine (The) [124]. Foods rich in L-tyrosine include dairy products, omega-3-rich foods, eggs, meat, fruits, vegetables, and nuts;white eggs, chicken, liver, beef, milk, and soybeans are a good source of L-phenylalanine [125]. The WHO dietary recommendation (2007) for L-phenylalanine is set at 25 mg/kg per day (with no TYR) [126]. Both L-tyrosine and L-phenylalanine are classified as neutral amino acids and are transported by the neutral amino acid transporter B^0^AT1 (encoded by SLC6A19 gene) across the apical membrane of intestinal epithelial cells. The transport is sodium-dependent and chloride-independent. B^0^AT1 is present along the small intestine and absent in the large intestine [127]. Further, L-phenylalanine is converted by phenylalanine hydroxylase (PAH, EC 1.14.16.1) into L-tyrosine. L-tyrosine is converted by tyrosine hydroxylase (TH, tyrosine 3-monooxygenase, EC 1.14.16.2) into L-dihydroxyphenylalanine (L-DOPA). DOPA-decarboxylase (DDC, aromatic L-amino acid decarboxylase, EC 4.1.1.28) converts L-DOPA into active DA. After its release, DA can either be taken up again by the presynaptic terminal (via the dopamine transporter, DAT, and/or by the plasma membrane monoamine transporter, VMAT2), or it can be broken down into inactive metabolites by monoamine oxidase (MAO, EC 1.4.3.4), catechol-O-methyltransferase (COMT, EC 2.1.1.6), and aldehyde dehydrogenase (ALDH, E.C. 1.2.1.3) [124].

In the GI tract, DA can be produced by the enteric neurons and non-neuronal tissues, including epithelial and immune cells [123], as well as gut bacteria [128]. Transcripts encoding DAT were found to be expressed along the adult murine gut, and the presence of TH and DAT immmunopositive neuron bodies (not just nerve fibers) within adult murine ENS plexuses was reported [129]. The presence of the intrinsic catecholaminergic (dopaminergic) phenotype in the human gut was also confirmed [130]. What is more, studies have demonstrated the presence (at mRNA and protein levels) of all five classes of dopamine receptors in the adult and fetal murine GI tract [131,132]. D2 receptors were present only on neurons, and D4 receptors were exclusively present on the mucosal layer [132]. The transcript expression and protein levels of D1A receptor were present in the rat gastroesophageal junction, stomach, pylorus, small intestine, colon, epithelial and muscle layers, and blood vessels and lamina propria cells [133]. Both the mRNA and proteins of D1, D2, and D5 receptors (D1R > D5R > D2R) were found in muscle fibers of the human lower esophageal sphincter [134]. The presence of D1R (D1A) was revealed in the apical membrane of villi [135] and at the base of the intestinal crypts of rat small intestine [136], while D5R (D1B) were present at both the apical and basolateral sides of the Brunner’s glands and intestinal crypts; D2R were distributed in the apical membrane of cells in both the Brunner’s glands and intestinal crypts [135]. The transcripts of all DAergic receptors have been identified in mucosal layers of the human colon [137]. Dopamine receptors are a class of G protein-coupled receptors with DA as the primary endogenous ligand, yet DA has a 10 times higher affinity for D5R than for D1R and a 20 times higher affinity for D3R than for D2R. D1R and D5R couple to the G protein Gs and activate adenyl cyclase, while receptor subtypes that belong to the D2-like subfamily inhibit adenyl cyclase and activate K^+^ channels [138].

The GI DAergic system was reported to modulate mucosal cytoprotection through bicarbonate secretion, blood flow regulation, and fluid absorption, as well as GI motility and inflammation (this is comprehensively reviewed in [139,140,141,142]).

### 3.2. Part 2—A Systematic Review

The search yielded limited data: eighteen studies were included into the qualitative analysis ([120,143,144,145,146,147,148,149,150,151,152,153,154,155,156,157,158,159] Table 3), which underlines the need to further experimentally explore the role of the GI RAS and DAergic systems in the regulation of intestinal permeability and their direct influence on tight junctions.

#### 3.2.1. Coexpression and Cosynthesis Studies on Epithelial DAergic System and RAS in the Intestines

Transcriptome analysis revealed that the human small intestine expressed the highest, among 61 human tissues and cell types, levels of ACE2, DDC, and SLC6A19. At the same time, immunohistochemical analysis confirmed the protein synthesis in small intestinal enterocytes. However, TH mRNA levels were below the detection threshold in those samples. Yet importantly, ACE2, DDC, and SLC6A19 genes did not belong to the molecular signature of human colonic or rectal cells [143].

Aging in rats was associated with increased DA levels, as well as TH and DAT expression in homogenized proximal colonic tissue. A decrease in D2R expression (at mRNA and protein levels) caused an increase in the D1/D2 ratio. Treatment with candesartan (an AT1R antagonist) induced an increase in the expression of D1R and D2R in aged rats (at mRNA and protein levels). Aging was also associated with an increased AT1R and decreased AT2R expressions (at mRNA and protein levels) [144].

Mice D1R knockouts exhibited a higher expression of AT1R only (at mRNA and protein levels) and increased DA levels. D2R mice knockouts exhibited a higher mRNA expression and protein level of AT1R, a lower mRNA expression and protein level of AT2R, and increased DA levels. AT1R mice knockouts exhibited a lower levels of D1R and D2R, yet with no changes in DA or noradrenaline (NA), in comparison with wild-type (WT) mice. Yet, aged AT1R deficient mice showed higher levels of D1R and D2R in comparison with WT aged mice. AT2R mice knockouts exhibited a higher expression of D1R and D2R (at mRNA and protein levels), as well as increased colonic DA levels. The levels of D1R in aged AT1R deficient mice treated with candesartan was higher than in young WT mice. However, D2R expression in aged AT1R-deficient, together with aged WT mice, was lower as compared to young WT littermates (at mRNA and protein levels). Aged AT1R knockouts exhibited a higher D1R and D2R levels [144]. Unfortunately, since those results reported changes in homogenates of the entire GI wall, they might not necessarily reflect on DAergic and RAS alterations directly in the colonocytes, not to mention enterocytes (bearing in mind the above-mentioned regionalization).

#### 3.2.2. The Influence of Intestinal DAergic System and RAS on Epithelial Permeability

Although the ACE2 knockout mice did not reveal any morphological alterations in the small and large intestine, the mice were prone to severe colitis when challenged with dextran sodium sulphate (DSS). The reaction was not due to the accumulation of Ang II (the deletion of AT1R did not rescue the severe DSS-induced colitis) or local haematopoietic cells (bone marrow transplantation from mutant mice into WT mice did not result in any obvious influence on DSS-induced colitis either). Yet, the serum levels of the neutral amino acids, including TYR and the essential amino acid tryptophan (TRP), were reduced in ACE2-deficient mice due to the lack of intestinal B^0^AT1, and indeed, a protein free-diet was reported to worsen DSS-induced colitis in WT mice to levels seen in ACE2 knockouts. What is more, the luminal ileocecal microbiome of ACE2-deficient mice was altered. Nicotinamide (tryptophan is required for its in vivo synthesis) was reported to alleviate DSS-induced colitis, and both diets rich in tryptophan and nicotinamide treatment reverted the composition of the intestinal microbiota of ACE2 knockout mice [147].

Colonic ACE2 activity was lower in the old male mice compared to young littermates, and MasR and AT1R levels were increased, while the ACE activity was indifferent. Plasma concentrations of FITC–dextran and plasma ZO-1 were higher in aging mice, yet after treatment with Ang (1-7), both were comparable with that observed in the younger mice. What is more, treatment with Ang (1-7) decreased the up-regulated mRNA levels of colonic ZO-1 and reshaped the architecture of the aging colonic epithelial layer—it restored the structural integrity of the epithelium, increased the mucus layer thickness and the number of goblet cells, and increased the synthesis of tight junction proteins—claudin 1 and occludin, as well as restructured the gut microbiome [145].

DA induced an increase in duodenal epithelial permeability in rats (SCH23390—a D1/D5R antagonist, but not sulpiride—a D2/D3R antagonist, was able to block the effect). D1R immunoreactivity was weakly distributed in the apical membrane of epithelial cells in villi and lamina propria, while a strong D5R immunoreactivity was observed in both the apical and basolateral sides of the duodenal villi. Transgenic D5S390G mice, which exhibited increased duodenal D5R immunoreactivity in comparison with WT mice, had increased FITC–dextran permeability, decreased TEER in duodenal preparations, and lower duodenal levels of ZO-1 and occludin (the claudin-1 level was indifferent). What is more, hyperenteric DA rats characterized by enhanced enteric DA exhibited increased FITC–dextran permeability. Those rats were characterized by an impaired duodenal mucosal barrier similar to that observed in transgenic D5S390G mice and were characterized by increased D5R (but not D1R) protein synthesis in duodenal preparations, strong D5R-immunoreactivity in the Brunner’s glands and duodenal villi, and a lower synthesis of TJ proteins, i.e., ZO-1 and occludin (but not claudin-1). The presence of D5R immunoreactivity was confirmed in the human duodenal epithelium [146].

#### 3.2.3. Intestinal B^0^AT1 and RAS

In the human intestine, B^0^AT1 colocalized with ACE2 along the brush border membrane of the duodenum and terminal ileum enterocytes on the villi. B^0^AT1 was not detected in the colon, yet ACE2 was present within colonic crypts. The mRNA of B^0^AT1 was more abundant in small as compared to large intestine. The mucosal level of the ACE mRNA was higher in the terminal ileum, while the level of ACE2 mRNA was comparable along the small intestine. What is more, patients treated with ACEIs had increased duodenal mucosal ACE2 and B^0^AT1 mRNA levels in comparison with nontreated controls. No differences were found in patients treated with ARBs. And the gene expression of duodenal ACE2 was correlated with that of B^0^AT1 [120].

The cosynthesis of human or mouse ACE2 with B^0^AT1 resulted in increased B^0^AT1 activity caused by an increased synthesis of B^0^AT1 on the X. laevis oocytes surface, which was ACE2-dependent. Moreover, the copresence of both proteins was observed in the apical membrane of the human small intestine, both in the jejunum and ileum. However, ACE2 was able to hydrolyze peptides regardless of the cosynthesis transporter generating a local concentration of amino acids, which then became substrates of amino acid transporters, such as B^0^AT1 [150].

ACE2 was coimmunoprecipitated with B^0^AT1 from the intestinal brush border membranes of WT mice. Furthermore, B^0^AT1 in the intestinal brush border was completely absent in mice lacking ACE2 (but normally expressed in the kidneys) [149]. Thus, the amount of all neutral amino acids was increased in the ileal lumen of ACE2 null mice, including TYR and PHE. Interestingly, a low-protein (7% casein/1.2 mg/kg niacin) diet resulted in either decreased or increased (including TYR and PHE) plasma levels of neutral amino acids in ACE2 null mice compared to WT littermates. Normal protein (20% casein/30 mg/kg niacin) intake resulted in lower plasma levels of TYR and PHE in ACE2 null mice in comparison to WT mice [148].

#### 3.2.4. Intestinal Na^+^/K^+^ ATPase Activity

In rats, ouabain (Na^+^/K^+^ ATPase inhibitor) treatment (intraperitoneally for 4 days) was associated with an increased jejunal synthesis of claudin-1, -3, and -5 (but not claudin-2 and -4), while in the colon, it was associated with decreased claudin-3 levels. Ouabain pretreatment prevented LPS-induced intestinal barrier damage assessed as the TEER and paracellular flux of sodium fluorescein in jejunal and colonic fragments [151]. And these results suggest a functional relationship between intestinal Na^+^/K^+^ ATPase activity and TJ assembly.

Ang II enhanced the activity of microsomal Na^+^/K^+^ ATPase in the mucosal scrapings of rat colons taken from animals that were nephrectomized 24 h before [159].

DA produced a decrease in Na^+^/K^+^ ATPase activity in jejunal enterocytes from 20-day-old rats, and the effect was antagonized by SKF-83566 (a selective D1/D5R antagonist), but not sulpiride, and was mimicked by SKF-38393 (a selective D1/D5R partial agonist), but not by quinerolane (a D2/D3R agonist) [153,156].

Na^+^/K^+^ ATPase activity was decreased in jejunal enterocytes isolated from 20- but not 40-day-old rats on a high-salt intake, and the effect was blocked with benserazide (DDC inhibitor) pretreatment. The jejunal cell plasma membrane exhibited the presence of only the Na^+^/K^+^ ATPase α1-isoform. And plasma membranes isolated from jejunal cells from 20-day-old rats on a high-salt intake showed a reduction in α1 subunit abundance compared with age-matched controls receiving normal saline, whereas in 40-day-old animals, high salt did not affect the α1 subunit abundance. The jejunal basal levels of L-DOPA, DA, and NE in the mucosa of 20-day-old rats were higher than in 40-day-old rats. High-salt intake increased DA (but not NE) tissue levels in 20-day-old rats only. Benserazide treatment increased L-DOPA levels and abolished the increase in DA after high-salt intake. In 40-day-old rats, high-salt intake did not affect DA level, benserazide pretreatment increased L-DOPA levels, and DA did not inhibit Na^+^/K^+^ ATPase activity [157].

DA caused a concentration-dependent decrease in TEER in jejunal preparations from 20- and 60-day-old rats. Na^+^/K^+^ ATPase activity in isolated jejunal enterocytes from 60-day-old rats was higher yet unresponsive to DA [156].

Low-sodium intake for 2 weeks increased DA levels in the jejunal mucosa and decreased L-DOPA tissue levels in 60-day-old rats, while high-sodium intake for 2 weeks did not change their levels. In 60-day-old rats fasted for 72 h, the effect of refeeding for 24 h with a low-sodium intake failed to change DA levels but increased the levels of L-DOPA, while refeeding for 24 h with high-sodium intake increased the tissue levels of both DA and L-DOPA. Jejunal Na^+^/K^+^ ATPase activity in rats fasted for 72 h followed by 24 h intake of normal and high-sodium was lower than in rats fed with the same diets for 2 weeks. Prolonged low sodium intake decreased jejunal Na^+^/K^+^ ATPase activity. In rats on 2 weeks of high-sodium intake, ouabain was found to be more potent in reducing jejunal TEER than in rats with normal and low-sodium intake. DA did not alter Na^+^/K^+^ ATPase activity or TEER in jejunal epithelial preparations, either in rats on 2 weeks or 24 h of normal, low-, or high-sodium intake [153].

The basal levels of mucosal DA in 20-day-old rats were twofold higher than in 40-day-old rats. Both age groups increased mucosal DA production after high-salt intake. The NE levels were much higher in 20- than in 40-day-old rats, but the NE content was indifferent after high-salt intake in all groups. In 20- but not in 40-day-old rats or 80-day-old rats, a lower net sodium absorption was observed after high-salt intake compared to age-matched controls on normal salt intake. The inhibition of DA synthesis with benserazide increased the net sodium absorption in younger rats on high-salt intake compared to untreated younger rats on high-salt intake. In fact, the values for net sodium absorption in benserazide-treated 20-day-old rats on high-salt intake were similar to those of age-matched controls on normal salt intake [158].

The tissue levels of DA or NA did not differ in 12-week-old rats either on low-, normal, or high-sodium intake. DA and SKF38393, but not quinerolane, reduced jejunal Na^+^/K^+^ ATPase in rats on a low-sodium diet only. The inhibitory effect of DA upon jejunal Na^+^/K^+^ ATPase activity was prevented by SKF-835660, while the D2R antagonist failed to revert the effect of the DA. A change from low-, normal, or high-sodium intake increased basal jejunal Na^+^/K^+^ ATPase activity, which was accompanied by an attenuation of the inhibitory effect of DA. The unresponsiveness of jejunal Na^+^/K^+^ ATPase activity to DA in rats on either normal or high-sodium intake may not be related to an increased salt intake but to decreased food (protein) intake vs. rats on low-sodium intake [154].

The basal Na^+^/K^+^ ATPase activity in jejunal enterocytes from 6-month-old rats was higher compared to 24-month-old rats. High-sodium intake for 24 h decreased the intestinal Na^+^/K^+^ ATPase activity in jejunal enterocytes from 6-month-old rats only. DA did not change the Na^+^/K^+^ ATPase activity in rats on either normal or high-sodium intake. The levels of L-DOPA and DA in the jejunal mucosa of 6-month-old rats were higher compared to 24-month-old rats on normal sodium intake; however, the latter had higher DDC activity, which correlated with their DA/L-DOPA tissue ratios. High-sodium intake failed to alter the levels of L-DOPA or DA regardless of age. However, high-sodium intake reduced DA/L-DOPA tissue ratios (with a parallel reduction in DDC levels) in 24-month-old rats. The tissue levels of NA were similar in both groups of rats regardless of sodium intake [152].

Unfortunately, all the above-mentioned experiments were performed on male rats only.

## 4. Discussion

### 4.1. Summary of Evidence

The existing evidence, although limited and indirect, points towards the existence of the mutual crosstalk between the GI renin–angiotensin and DAergic systems in the regulation of intestinal permeability by tight junctions. The GI tract, pancreas, and spleen are known to be the major sources of DA production in the body; still, an understanding of the distribution of DA receptors along the GI tract and the exact role of the epithelial (non-neuronal) DAergic system is incomplete. Furthermore, the presence of enteric (intrinsic) DAergic neurons remain debatable (at least by some authors), and replication studies should be appreciated. Experimental evidence suggests that DA, L-DOPA, and DDC levels in the intestinal epithelium exhibit age and regional differences [144,152,157,158], yet no data are available with regard to sex differences, and foremostly, data from human subjects of different age and sex groups remain insufficient [143]. DA via D5R could be responsible for an impaired duodenal mucosal barrier with decreased epithelial ZO-1 and occludin levels (but not claudin-1) [146]. On the other hand, it was also reported that D5R signaling could be protective against DSS-induced colitis in mice. D5R deficiency exacerbated experimental colitis, and furthermore, SKF-38393 treatment was unable to attenuate the clinical signs of colitis in D5R-deficient mice compared to WT littermates [160]. GI DAergic imbalance could also impact neurotransmission and neuronal fate in the central nervous system, with long-lasting consequences such as neuropsychiatric and neurodegenerative disorders [161]. Collectively, such results imply the complex and heterogeneous role of DA and its receptors along the GI tract and underline the need and importance to address those issues.

Susceptibility to severe DSS-induced colitis in mice was also characteristic for ACE2-deficient mice lacking intestinal (but not renal, which was still synthesized) B^0^AT1 [147,149]. The synthesis in the apical membrane of enterocytes and activity of the B^0^AT1 sodium-dependent neutral amino acid transporter was indeed reported to be ACE2-dependent [120,148,149,150,162]. Thus, the amount of all neutral amino acids was reported to be increased in the ileal lumen of ACE2 null mice, including aromatic amino acids such as TYR and PHE, and was decreased in the plasma [148]. A protein-free diet was reported to worsen DSS-induced colitis in WT mice to levels similar to ACE2 knockouts, while nicotinamide (tryptophan is required for its in vivo synthesis) was reported to alleviate DSS-induced colitis in ACE2 knockout mice [147]. Interestingly, patients treated with ACE inhibitors (ACEIs) had increased duodenal mucosal ACE2 and B^0^AT1 mRNA levels in comparison to nontreated controls [120]. Recently, a growing number of studies underline the unique role of dietary amino acids in the regulation of intestinal epithelial cells homeostasis [163], as well as in alleviating and preventing intestinal inflammation [164]. For instance, it was reported that PHE could exert anti-inflammatory action on indomethacin-induced inflammatory bowel disease in rats [165]. Aromatic amino acids, including TRP, PHE, and TYR, were reported to attenuate intestinal inflammation through the activation of calcium-sensing receptors in pigs [166]. Furthermore, mice exposed to radiation and treated with an amino acid-based oral rehydration solution (a mixture of threonine, valine, serine, tyrosine, and tryptophan) displayed enhanced intestinal epithelial proliferation with decreased paracellular permeability [167]. TRP alike PHE is an essential amino acid; yet, it has low tissue storage, and its overall concentration in the body is the lowest among all amino acids. In the bloodstream, TRP competes with other neutral amino acids (including PHE and TYR) for the blood–brain transporter [168]. TRP is known to be a poor substrate of the B^0^AT1 transporter [127]; still, the ACE2:B^0^AT1 complex plays a predominant role in TRP absorption from the intestinal lumen to the cytosol of enterocytes [147], and thus, the defective SLC6A19 gene has been especially associated with TRP malabsorption [169]. It was also reported that the dietary depletion of TRP, TYR, and PHE decreased serum and hippocampal levels of these amino acids and further altered serotonin and monoamines (and their metabolites) concentrations in different brain regions in mice. The dietary restriction reduced the relative abundance of the fecal genus Roseburia, and foremostly, a negative correlation between the relative percentage of the fecal genus Enterococcus and fecal TYR concentrations was found [170]. Interestingly, DOPA decarboxylasing and DA dehydroxylasing bacteria belong to genus Enterococcus [171], which further implies the complexity of the GI epithelial DAergic system. Moreover, the use of germ-free animal models has provided evidence to support the idea of early life gut colonization as critical to brain development and DAergic neurotransmission in particular [172], which in turn implies the key role of dietary patterns in shaping the microbiome-gut–brain axis.

What is more, the ileocecal microbiome of ACE2-deficient mice lacking intestinal B^0^AT1 was reported to be altered too [147]. Thus, the ACE2:B^0^AT1 functional unit is not only important for multiple gut lumen activities in the rodent and human small intestine [120,147,150], including the metabolism of nutritive and bioactive peptides, as well as the absorption of sodium and organic nutrients [173], but should also modulate the microbiota–gut–brain axis. Unlike B^0^AT1, ACE2 is expressed along the small and large intestine (Table 2) [86,103,106,112,114,121,128]. In rats, aging was associated with a decrease in ACE2 levels and its activity, which was associated with fibrogenic and proinflammatory intestinal conditions [174]. However, in aging mice, treatment with Ang (1-7) decreased the upregulated mRNA levels of colonic ZO-1, restored the structural integrity of colonic epithelium, and the increased levels of epithelial claudin-1 and occludin [145]. The administration of Ang 1–7 also alleviated DSS-induced colitis in mice [174,175]. Still, based on animal models and retrospective studies in humans, the use of either angiotensin-converting enzyme inhibitors or Ang II receptor blockers was reported to be inconclusive (reviewed in [176]).

An aging-associated shift towards the proinflammatory ACE/ATR1 axis of the RAS was also associated with an increased colonic D1R/D2R ratio, a decreased D2R level, and higher levels of DA, TH, and DAT, which resembled the phenotype of D1R- and D2R-deficient mice with an increased AT1R/AT2R ratio and DA levels [144]. Yet, it remains unknown if the same interactions should be expected in the epithelial cells compared to all layers of the murine colon. It was reported that the basal levels of mucosal DA in 20-day-old rats were twofold higher than in 40-day-old rats and were increasing after high-salt intake [158]. What is more, it was reported that the levels of L-DOPA and DA in the jejunal mucosa of 6-month-old rats were higher compared to 24-month-old rats, and the latter had higher DDC activity [152]. It is noteworthy that DA exhibits the highest affinity towards D3R and D5R and the lowest towards D1R [129]. Low-affinity dopamine receptors (D1R and D2R), which are stimulated by high DA levels (1–10 μM), induced anti-inflammatory effects in the immune system [134,177]. D2R signaling, which should be activated by 1–5 μM DA, was impaired following gut inflammation (similar to aging [144]) and promoted the suppressive activity of regulatory T cells, as well as reduced vascular permeability [134]. The renal DAergic system opposes the antinatriuretic activity of the RAS by downregulating AT1R, upregulating AT2R and inhibiting ROS generation [135]. The stimulation of the AT1R increased Na^+^/K^+^ ATPase activity, while the AT2R was shown to inhibit Na^+^/K^+^ ATPase activity in isolated rabbit renal proximal tubule cells [178,179]. Yet, the relationship in the GI tract seems to be much more complex. While Ang II enhanced the activity of Na^+^/K^+^ ATPase [159], DA (and SKF-38393) decreased its activity only in jejunal enterocytes from 20-day-old rats [153,156] or rats on a low-sodium diet [154]. Furthermore, basal Na^+^/K^+^ ATPase activity in jejunal enterocytes was age- and diet-dependent [152,153,154,155,156,157].

Numerous drugs acting on the renin–angiotensin and DAergic systems are widely in use (examples are given in Table 4 and Table 5), yet their influence on the small and large intestinal barrier remains unmarked and experimentally neglected. The above-mentioned complexity of the GI DAergic and renin–angiotensin systems may also explain inconclusive attempts for drug repurposing in inflammatory bowel diseases [141,176,180]. The modulatory effect of intestinal microbes and/or the concomitant use of antibiotics and/or nutritional failures might partly explain their limited efficacy. On the other hand, given their wide clinical use, special attention should be given to their capacity to shape the intestinal mucosal barrier, which could have yet unrecognized and long-lasting systemic consequences, especially if given in combination with other medications.

### 4.2. Limitations

Studies included into the systematic analysis exhibited a high degree of variability and lacked the uniformity necessary to conduct a meta-analysis. Human studies addressing epithelial barrier integrity, together with renin–angiotensin and dopaminergic systems, are in high demand. What is more, insufficient and inconclusive findings of this current study also highlight the need to include the context of microbial diversity, age, and sex in all future studies involving both animal models and human subjects to deepen our understanding of the complex relationship between the GI renin–angiotensin and DAergic systems in the regulation of intestinal permeability.

## 5. Conclusions

Recently, intestinal barrier integrity has been recognized as significantly important to overall health and lifespan [181]. Still, more mechanistic studies are needed to fully comprehend the complex relationship between the renin–angiotensin and dopaminergic systems in the regulation of epithelial tight junctions. Some of the above-mentioned studies were conceptually and methodologically distinct or fragmentary, and none of them addressed sex differences. Due to the limited availability of experimental data, replication studies would be welcomed too. Transcriptomic and immunohistochemical analysis confirmed the copresence of DDC and ACE2 in the enterocytes [143], which highlight the RAS and DAergic synthetic and metabolic capacity of IECs. D5R-mediated action of DA, together with Ang (1-7), should be able to affect intestinal barrier structure and integrity [145,146]. Both the renin–angiotensin and dopaminergic systems influence intestinal Na^+^/K^+^ ATPase activity [151,152,153,154,155,156,157,158,159] and thus maintain electrolyte and nutritional homeostasis [153,154,155]. Moreover, B^0^AT1 and ACE2 colocalization indicates the direct role of the RAS in amino acid absorption, including the absorption of DA precursors [120,148,149,150]. Future studies should assess in the first instance (1) the structural and functional interaction between TJ-associated proteins and the GI renin–angiotensin and dopaminergic systems; (2) if deficiencies of specific amino acids could alter the assembly of TJs and the activity of the Na^+^/K^+^ ATPase throughout the GI tract; and (3) how age, reproductive hormones, and microbial metabolites influence the intestinal epithelial relationship between the renin–angiotensin and dopaminergic systems. Such detailed knowledge could ease drug repositioning and our understanding of the lack of treatment efficacy or long-lasting side effects of compounds acting on the dopaminergic and renin–angiotensin systems (or even their combination), which are already available on the market.

## Figures and Tables

**Table 1:** Tight junctions-associated proteins.

Class of TJ Protein	Type of Protein	Molecular Mass	Major Function	References
**Integral (intrinsic) transmembrane**	Occludin (OCLN, BLCPMG, PPP1R115, PTORCH1)	~65 kDa	barrier properties, expression of glucose transporters, activation of transcription factors	[31,32]
	Claudin (CLDN) 1–27	21–34 kDa	selective permeability—charge and size selectivity	[2,32,33,34]
	Junctional adhesion molecules (JAMs)	~40 kDa	barrier and cell motility, polarity, and proliferation	[32]
	Tricellulin (MARVELD2, MARVEL domain containing 2)	~66–72 kDa	formation of the epithelial barrier	
**Peripheral (extrinsic plaque) membrane protein**	Zonulin 1, 2 and 3 (ZO-1, 2, and 3 OR Tight junction protein-1, 2, and 3)	~47 kDa	a primary barrier—diffusion of solutes and a fence, maintenance of polarity	[35,36]
	Cingulin (CGN)	140–160 kDa	adaptor protein	[37,38]
	Paracingulin (Cingulin-like 1 protein, CGNL1, or JACOP)	~160 kDa		

**Table 2:** Human and rodent gastrointestinal renin–angiotensin system.

RAS Components	GI Distribution		References
	Quantification of Transcript Levels	Quantitative and Qualitative Protein Abundance	
Angiotensinogen	stomach, colon, and mesentery (r); enterocytes of jejunum and ileum (r); jejunal muscle wall (h); ileum (h); jejunum and colon (r); sigmoid and ascending colon (h)	antral and corporal gastric mucosa (WB, h); resident mesenchymal cells in the lamina propria, and vascular endothelial cells in antral and corporal gastric mucosa (IHC, h); small intestinal brush border membrane and lamina propria, muscularis mucosa, the muscle layer, and submucosal blood vessels (IHC, r); enterocytes of ileum and jejunum (WB, r),	[96,97,98,99,100,101,102,103]
Angiotensin I	N/A	colonic mucosa (RIA; h)	[104]
Angiotensin II	crypt and crypt–villus junction small intestinal epithelial cells (m)		[104,105]
Angiotensin (1–7)	jejunal enterocytes (r)	jejunal enterocytes (WB, r)	[106]
AT1R	esophageal mucosa (h); small intestinal epithelial brush border (m), enterocytes of jejunum and ileum (r); jejunal muscle wall (h); jejunum and colon (r); colon mucosa (h); sigmoid colon and ascending colon (h); proximal and distal colon (m); colon (r)	esophageal mucosa (WB, h); esophageal mucosa epithelium and stratum superficiale and spinosum, blood vessel walls supplying the epithelium and the lamina propria (IHC, h); antral and corporal gastric mucosa (WB, h); antral and corporal gastric mucosa—basal parts of most epithelial cells, endothelial cells of vessels, some resident mesenchymal cells in the lamina propria, and only antral mucosal endocrine cells (IHC, h); enterocytes of jejunum and ileum (WB, r); jejunum and entire ileum villus length at both brush border and basolateral membranes, lamina propria, muscularis mucosa, muscle layers, and submucosal blood vessels (IHC, r); jejunal myenteric plexus and musculature (IHC, h); muscularis of duodenum, jejunum, ileum, and colon (autoradiography, r); duodenum and ileum (especially longitudinal smooth muscle; autoradiography, r); mucosa, and the muscularis of jejunum, ileum, and colon (autoradiography, r); sigmoid colon and ascending colon vessel walls, surface epithelium, crypt bases, mesenchymal cells in the lamina propria (especially macrophages), and myofibroblasts (IHC, h)	[97,98,99,100,101,103,105,106,107,108,109,110,111,112,113,114]
AT2R	esophageal mucosa (h); enterocytes of jejunum and ileum (r); jejunal muscle wall (h); sigmoid colon and ascending colon (h); distal colon (m); colon (r)	esophageal mucosa (WB, h); esophageal mucosa epithelium and in superficial stratum and spinosum, as well as in blood vessel walls supplying the epithelium in the lamina propria (IHC, h); antral and corporal gastric mucosa (WB, h); antral and corporal gastric muscosa—basal parts of most epithelial cells, endothelial cells of vessels, and some resident mesenchymal cells in the lamina propria (IHC, h); jejunal myenteric plexus (IHC, h); enterocytes of jejunum and ileum (WB, r); entire jejunum villus length at the brush border membrane (IHC, r); mucosa and muscularis of jejunum, ileum, and colon (autoradiography, r); sigmoid colon and ascending colon—mesenchymal cells and parts of surface epithelium, as well as a small number of crypt cells (IHC, h);	[97,98,99,100,101,103,104,109,111]
MasR	muscular layer of distal esophagus (h); jejunal enterocytes (r); ileum (m); jejunum and colon (r); colon mucosa (h); colon (r)	jejunal enterocytes (WB, IHC, r); ileum—with a neuron-specific pattern (IHC, m); colon mucosa (IHC, h)	[103,104,105,106,112,114,115,116]
Renin	muscular layer of distal esophagus (h); small intestine (m, r, h); sigmoid colon and ascending colon (h)	antral and corporal gastric mucosa (WB, h); resident mesenchymal cells in the lamina propria and vascular endothelial cells in antral and corporal gastric mucosa (IHC, h); sigmoid colon and ascending colon—surface epithelium, vessel walls, muscularis mucosae, mesenchymal cells in the lamina propria (IHC, h)	[97,101,116,117]
ACE	esophageal mucosa (h); duodenum, jejunum, ileum (h); jejunal muscle wall (h); duodenal and ileal mucosa (h); enterocytes of jejunum and ileum (r); jejunum and colon (r); sigmoid colon and ascending colon (h); colon mucosa (h); colon (r)	esophageal mucosa (WB, h)—the capillary walls located at the tip of the papillae and in the blood vessel walls in the lamina propria (IHC, h); stomach fundic chief cells, and the mucin-secreting cells of the antral and pyloric region (IHC, h); antral and corporal gastric mucosa (WB, h); vascular endothelial cells in both the antral and corporal gastric mucosa (IHC, h); entire villus length at the brush border membrane of the jejunum (IHC, r); enterocytes of jejunum and ileum (WB, r); small intestinal epithelial cells, microvilli, brush borders, and microvascular endothelium (IHC, h); mucosa and muscularis of duodenum, jejunum, ileum, and colon (autoradiography, r); jejunum and colon (WB, r); vessel walls and mesenchymal cells in lamina propria and submucosa and weakly in parts of the surface epithelium in ascending colon (IHC, h)	[86,97,98,99,100,101,103,107,111,112,114,118,119,120]
ACE2	jejunal enterocytes (r); duodenum, jejunum, ileum, caecum, and colon (h); duodenal and ileal mucosa (h); jejunum and colon (r); colonic mucosa (h); colon (r)	jejunal enterocytes (WB, r); small intestinal enterocytes (brush border), smooth muscle cells and endothelium of vessels from the stomach, small intestine and colon, smooth muscle cells of the muscularis mucosae and the muscularis propria (IHC, h); along the villi and in the crypts of duodenum and terminal ileum, as well as in crypts in ascending colon (IHC, h); jejunum and colon (WB, r)	[96,103,106,112,114,120,121]

Abbreviations: IHC—immunohistochemistry, h—human samples, N/A—not available, m—murine samples, r—rat samples, RIA—radioimmunoassay, WB—Western blotting.

**Table 3:** All studies included in the systematic qualitative analysis conducted according to the Preferred Reporting Items for Systematic reviews and Meta-Analyses (PRISMA) methodology [16].

References	Animal Model(s)	Ex Vivo Study (ies)	Human Sample (s)	Major Conclusions
**Co-expression and co-synthesis studies on epithelial DAergic system and RAS in the intestines**				
Nataf and Pays 2021 [143]	X	X	Enterocytes of the small intestine	ACE2 coregulated not only with DDC but also with other genes involved in DA/trace amines metabolic pathway and the absorption of microbiota-derived L-DOPA, as well as in neutral amino acids serving as precursors to neurotransmitters.
Garrido-Gil et al., 2018 [144]	Male (2- to 3-month-old and 18- to 20-month-old) Sprague Dawley rats and C57/BL6 mice—D1 or D2 receptor knockout and AT1 or AT2 receptor knockout	X	X	Aged rats showed a decrease in colonic dopamine D2 receptor, an increase in AT1 receptor, a decrease in AT2 receptor expression and synthesis, and exhibited increased levels of colonic DA and NA.
**The influence of intestinal DAergic system and RAS on epithelial permeability**				
Chittimalli et al., 2023 [145]	C57BL/6 male (3–7-month-old) mice	Ogranoids from mouse colons	X	Ang (1–7) restored gut barrier integrity and increased levels of claudin 1 and occludin in the aging colon.
Feng et al., 2017 [146]	Male Sprague Dawley rats (210–240 g), the rat model of hyperendogenous enteric DA, C57BL/6J mice (20–25 g), transgenic mice with differentially mutated D5R	X	Duodenal mucosa	DA altered duodenal permeability via D5R.
Hashimoto et al., 2012 [147]	Wild-type and ACE2 knockout mice treated with DDS	X	X	Deficiency in murine ACE2 resulted in highly increased susceptibility to intestinal inflammation induced by epithelial damage.
**Intestinal B^0^AT1 and RAS**				
Vuille-dit-bille et al., 2015 [112]	X	X	Mucosal biopsies from four different parts of the gastrointestinal tract from patients treated with either ACE inhibitors, AT1-receptor blockers, or controls.	Increased intestinal mRNA levels of ACE2 and B^0^AT1 were noted in samples from patients treated with ACE inhibitors.
Singer et al., 2012 [148]	Wild-type and ACE2 knockout mice on a standard diet or a low-protein/low-niacin diet for up to 85 days	Proximal small intestine rings	X	All neutral amino acids required functional B^0^AT1 to be efficiently absorbed along the small intestine.
Camargo et al., 2009 [149]	Wild-type and ACE2 knockout male mice	Brush border membrane vesicles from wild-type and ACE2 knockout murine small intestine	X	B^0^AT1 in small intestine was dependent on ACE2.
Kowalczuk et al., 2008 [150]	X	X. laevis oocytes	Jejunum, ileum	B^0^AT1 and ACE2 coexpressed in the apical membrane of human jejunal and ileal enterocytes.
**Intestinal Na^+^, K^+^-ATPase activity**				
Markov et al., 2020 [151]	Male Wistar (180–230 g) rats	X	X	Chronic ouabain treatment differently affected claudin synthesis in jejunal and colonic enterocytes.
Vieira-Coelho et al., 2001 [152]	Adult (6 months) and old (24 months) Fischer 344 rats on normal and high-salt intake (24 h).	Rat jejunal epithelial cells	X	Intestinal dopaminergic tonus in old rats was higher than in adult rats and was accompanied by a lower basal intestinal Na^+^/K^+^-ATPase activity. High-sodium diet failed to alter intestinal dopaminergic tonus or Na^+^/K^+^ ATPase activity in old rats, whereas in adult rats, an increase in AADC activity was accompanied by decreased Na^+^/K^+^ ATPase activity.
Vieira-Coelho et al., 2001 [153]	Male Wistar rats (18- to 20-day-old)	Rat jejunal epithelial cells	X	DA significantly decreased Na^+^/K^+^ ATPase activity in rat jejunal cells, and the effect was antagonized by D1 receptor antagonist SKF 83566 but not affected by D2-like receptor antagonist S-sulpiride and was mimicked by D1-like receptor agonist SKF 38393, but not by D2-like receptor agonist quinerolane.
Lucas-Teixeira et al., 2000 [154]	Male 12-week-old spontaneous hypertensive rats (SHR) and Wistar Kyoto rats (WKR) on normal, low-, and high-sodium intake	X	X	Inhibition of jejunal Na^+^/K^+^ ATPase activity by D1 dopamine receptor activation was dependent on salt intake in WKY rats, and SHR animals failed to respond to DA, irrespective of their salt intake.
Lucas-Teixeira et al., 2000 [155]	Male (60-day-old) Wistar rats on normal, low-, and high-sodium diet	X	X	Prolonged low salt intake decreased activity of jejunal Na^+^/K^+^ ATPase and increased rate of DA synthesis (with significantly increased levels of DA and decreased of L-DOPA).
Vieira-Coelho et al., 2000 [156]	Adult (60-day-old) and young (18–20-day-old) male Wistar rats	Membrane preparations from rat intestinal mucosa; rat jejunal epithelial cells	X	Na^+^/K^+^ ATPase activity in isolated jejunal epithelial cells from adult rats was 2.4 fold that in young rats. Levels of DA in the jejunal mucosa were more than twice in young rats compared to the adult animals, and DA/NA tissue ratios in young animals were 7.5 fold those in adult animals.
Vieira-Coelho et al., 1998 [157]	Sprague Dawley rats aged 20 (weaning) and 40 (adult) days	Rat jejunal epithelial cells	X	In 20-day-old but not in 40-day-old rats Na^+^/K^+^ ATPase activity was significantly reduced during high-salt diet, and the inhibition was abolished by a blocker of DA synthesis. Decreased Na^+^/K^+^ ATPase activity was associated with a decrease in α1 subunit at the plasma membrane.
Finkel et al., 1994 [158]	Sprague Dawley rats, aged 20 (weanling), 40 (adolescent), and 80 (adult) days	X	X	Weanling animals had a greater jejunal sodium absorption than older animals. High-salt diet resulted in a decreased intestinal sodium absorption in weanling rats but not in adult rats, and an endogenous DA should play a role in this regulation.
Gutman et al., 1972 [159]	X	Mucosal scrapings from rat colon	X	Ang II increased Na^+^/K^+^ ATPase activity in colonic microsomes.

Abbreviations: ACE2: angiotensin-converting enzyme 2, B^0^AT1: neutral amino acids transporter, DA: dopamine, DDC: aromatic L-amino acid decarboxylase, NA: noradrenaline.

**Table 4:** Currently FDA approved drugs acting on renin–angiotensin system.

Indications	Generic Name	Target Site
Hypertension, Heart failure, Coronary artery disease, Myocardial infarction	Candesartan	Angiotensin II receptor blockers
	Valsartan	
	Losartan	
	Irbesartan	
	Eprosartan	
	Olmesartan	
Left ventricle hypertrophy, Chronic kidney disease, Hypertension, Diabetic nephropathy	Benzapril	Angiotensin converting enzyme inhibitors
	Captopril	
	Enalapril	
	Quinapril	
	Ramipril	
	Lisinopril	
	Perindopril	
	Fosinopril	

**Table 5:** Current FDA-approved drugs acting on dopamine receptors.

Indications	Generic Name	Receptors
**Agonists**		
Parkinson’s disease, Restless leg syndrome, Hyperprolactinemia	Apomorphine	D1, D2
	Bromocriptine	D1, D2
	Dihydroergocryptine	D1–D3
	Fenoldopam	D1
	Rotigotine	D1–D5
	Cabergoline	D2
**Antagonists**		
Schizophrenia, Bipolar disorders, Mania, Alcohol withdrawls, Depression	Chlorpromazine	D1–D5
	Haloparidol	D2–D4
	Amisulpride	D2, D3
	Aripiprazole	D2
	Olanzapine	D1–D5
	Ziprasidone	D1–D4
	Loxapine	D2–D4
	Risperidone	D2–D4

## Data Availability

No new data were created or analyzed in this study. Data sharing does not apply to this article.
